# Insights into the role of HIV-1 Vpu in modulation of NF-ĸB signaling pathways

**DOI:** 10.1128/mbio.00920-23

**Published:** 2023-07-06

**Authors:** Robert Z. Zhang, Melissa Kane

**Affiliations:** 1 Department of Pediatrics, Division of Infectious Diseases, University of Pittsburgh School of Medicine, Pittsburgh, Pennsylvania, USA; 2 RK Mellon Institute for Pediatric Research, UPMC Children’s Hospital of Pittsburgh, Pittsburgh, Pennsylvania, USA; 3 Center for Microbial Pathogenesis, UPMC Children’s Hospital of Pittsburgh, Pittsburgh, Pennsylvania, USA; University of North Carolina at Chapel Hill, Chapel Hill, North Carolina, USA

**Keywords:** NF-κB, Vpu, HIV-1, β-TrCP1

## Abstract

HIV-1 inhibits the activation of nuclear factor kappa-light-chain-enhancer of activated B cells (NF-κB) to prevent the induction of a proinflammatory state but also activates the NF-κB pathway to promote viral transcription. Thus, optimal regulation of this pathway is important for the viral life cycle. In recent work, Pickering et al*.* (3) demonstrate that HIV-1 viral protein U has contrasting effects on the two distinct paralogs of β-transducin repeat-containing protein (β-TrCP1 and β-TrCP2) and that this interaction has important implications for the regulation of both the canonical and non-canonical NF-κB pathways. Additionally, the authors identified the viral requirements for the dysregulation of β-TrCP. In this commentary, we discuss how these findings further our understanding of how the NF-κB pathway functions during viral infection.

## TEXT

**Fig 1:**
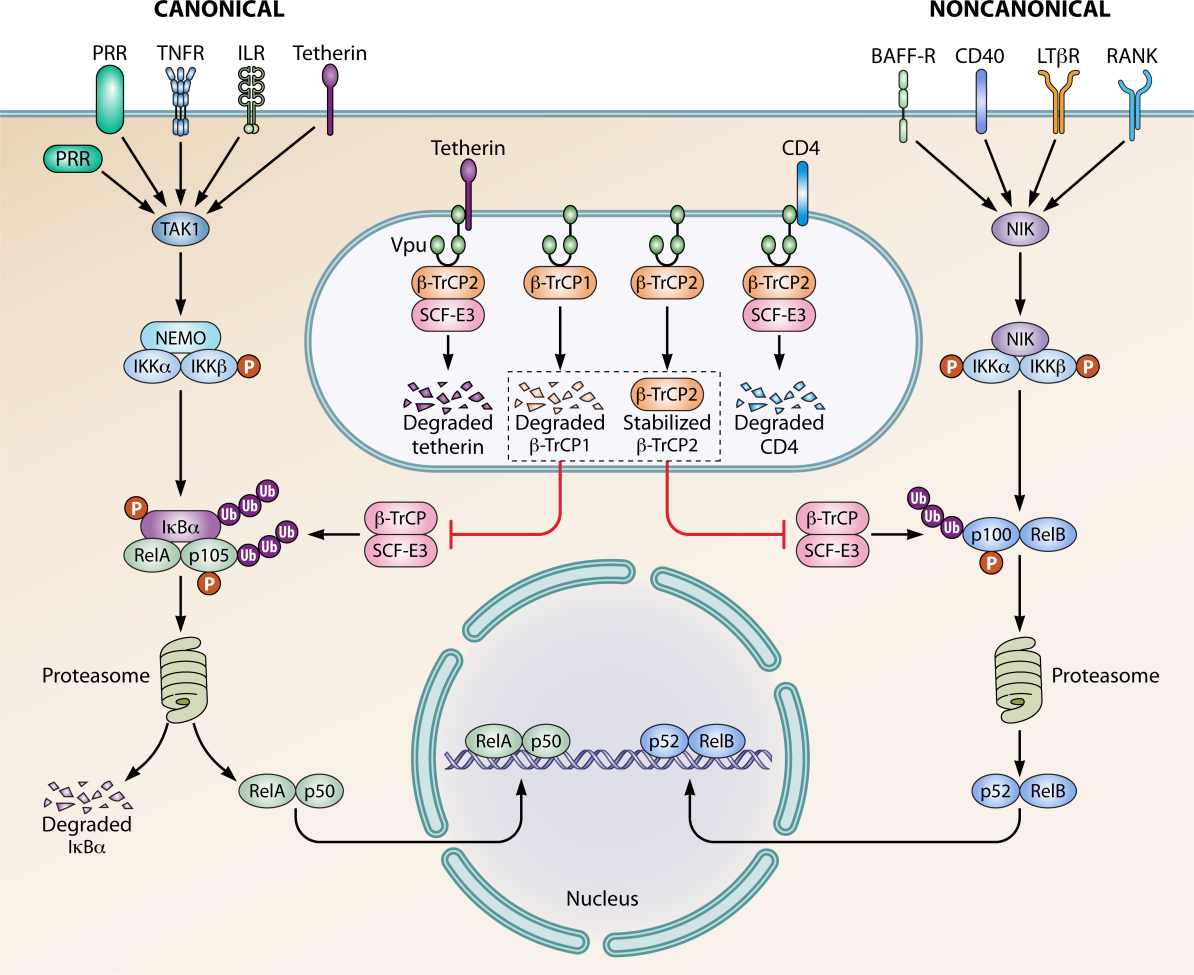
HIV-1 Vpu inhibits the NF-kB signaling pathway. Stimulation by various cytokines and receptors triggers signaling cascades that lead to the activation of the NF-κB pathway. The canonical NF-κB pathway is activated by a diverse group of stimuli such as tumor necrosis factor receptor (TNFR) superfamily members, various cytokine receptors, pattern recognition receptors (PRRs), and tetherin. Upon stimulation, transforming growth factor beta-activated kinase 1 (TAK1) is recruited to nuclear factor-κB essential modulator (NEMO) and activates the inhibitor of kappa B kinase (IKK) complex via phosphorylation of IKKβ. Activated IKKβ phosphorylates inhibitory kappa B alpha (IκBα) and p105, which leads to proteasomal degradation and processing by the β-TrCP substrate adaptor portion of an E3 cullin-RING ligase (SCF^β-TrCP^). Degradation of IκBα results in nuclear translocation of NF-κB complexes, which then regulate gene expression. In contrast, the non-canonical NF-κB pathway is triggered by a specific set of receptors, including a subset of TNFR superfamily members, interleukin receptors (ILR), CD40, lymphotoxin β receptor (LTβR), B-cell activating factor receptor (BAFF-R), and receptor activation of NF-κB (RANK). Stimulation leads to the activation of NF-κB-inducing kinase (NIK) and subsequent phosphorylation of IKKα. IKKα then phosphorylates p100 leading to the partial proteasomal degradation by SCF^β-TrCP^. NF-κB complexes are then translocated to the nucleus. Vpu binds CD4 and SCF^β-TrCP2^ to downregulate CD4 expression. Vpu antagonizes tetherin and targets tetherin for proteasomal degradation in an SCF^β-TrCP2^-dependent manner. Additionally, HIV-1 Vpu inhibits proteasomal processing of p100 and p105 in a process that requires both β-TrCP1 and β-TrCP2 to prevent NF-κB signaling.

Nuclear factor kappa-light-chain-enhancer of activated B cell (NF-κB) proteins are a family of highly conserved transcription factors that modulate multiple processes of the immune response. NF-κB activation occurs through two distinct signaling pathways, the canonical and the non-canonical pathway (Fig. 1). Activation of either NF-κB pathway results in the expression of proinflammatory genes on viral infection (1). Thus, viruses have evolved mechanisms to target multiple aspects of the NF-κB pathway. However, some viruses, such as HIV-1, have also evolved to co-opt this pathway. In fact, NF-κB is required for HIV-1 viral transcription (2), but the virus also encodes multiple accessory proteins that inhibit NF-κB signaling. Thus, viruses must maintain a delicate balance between inhibition and activation of NF-κB, and studying the mechanisms by which viruses are capable of modulating NF-κB may prove informative in the development of novel antivirals.

In recent work*,* Pickering et al. (3) focused on the regulation of NF-κB by the HIV-1 accessory protein viral protein U (Vpu), which has multiple roles during late viral replication. Using laboratory-adapted strains of HIV-1, previous reports have shown that Vpu promotes viral replication and dissemination by degrading CD4 to dissociate it from newly synthesized viral Env (4). Additionally, Vpu antagonizes the restriction factor tetherin to promote the release of viral progeny and to inhibit NF-κB signaling (5) (Fig. 1). However, as the authors correctly point out, previous studies using laboratory-adapted strains of HIV-1 are limited, they employed the use ofviral proteins from primary isolates to determine how viral proteins modulate NF-ĸB signaling during natural infection. Laboratory-adapted viral strains do not undergo the same selective pressures and as a result, viral proteins from these strains may have impaired activity when compared to viral proteins from primary isolates. Indeed, this group has previously shown that Vpu from a laboratory-adapted viral strain has suboptimal activity against tetherin (6). Accordingly, Pickering et al. begin with the logical hypothesis that Vpu from laboratory-adapted HIV-1 may also have diminished activity against NF-ĸB and that Vpu’s capacity to inhibit NF-ĸB may be underestimated. The authors use Vpu from the molecular clone of laboratory-adapted NL4.3 as well as a highly active primary Vpu, 2_87, which is representative of Vpu found in patients with natural subtype B infections.

To understand the role of primary Vpu in the inhibition of NF-κB, Pickering et al. measured the ability of Vpu to suppress NF-κB signaling induced by both canonical and non-canonical stimuli. While laboratory-adapted NL4.3 was capable of inhibiting both the canonical and non-canonical NF-κB pathways, the inhibition of both NF-κB pathways was more pronounced and sustained by primary Vpu. These findings highlight that the use of laboratory-adapted viruses is not always representative of natural pathogenesis. Furthermore, mutants of both Vpus that are unable to bind β-TrCP were defective in their ability to inhibit NF-κB activation, confirming previous reports that inhibition of both NF-κB pathways by Vpu is meditated by β-TrCP (7).

Previous work shows that HIV-1 Vpu sequesters β-TrCP in the cytoplasm to prevent the proteasomal degradation of inhibitory kappa B alpha (IκBα), resulting in the inhibition of the canonical NF-ĸB pathway (8). Interestingly, Pickering et al. make the key finding that primary Vpu simultaneously leads to the proteasomal degradation of β-TrCP1 and relocalization and sequestration of β-TrCP2 with Vpu in the perinuclear region. Existing literature suggests that Vpu may target β-TrCP1 for degradation by exploiting β-TrCP2 (9). But surprisingly, Pickering et al. demonstrate that the knockdown of β-TrCP2 has no effect on the reduced levels of β-TrCP1. Furthermore, the authors show that the knockdown of both β-TrCP1 and β-TrCP2 is required to phenocopy NF-κB inhibition by Vpu (Fig. 1). These findings implicate both β-TrCP1 and β-TrCP2 in controlling NF-ĸB signaling in a non-redundant fashion. It is still unclear how the two paralogs of β-TrCP cooperate to promote NF-κB signaling in the context of infection. Future work may reveal the mechanisms by which Vpu depletes β-TrCP1, and why this disparity between β-TrCP1 and β-TrCP2 is important for HIV-1 replication.

IκBα, p100, and p105 function as inhibitors in the NF-κB pathway. The SCF^β-TrCP^ E3 ubiquitin ligase regulates these inhibitors by targeting them for proteasomal degradation. Proteasomal degradation of IκBα and partial proteasomal degradation of p105 and p100 into p50 and p52, respectively, are required for NF-κB activity. To investigate how Vpu affects the NF-ĸB signaling pathway, Pickering et al. thoroughly examine components from both canonical and non-canonical pathways. The authors show that primary and laboratory-adapted Vpu leads to elevated expression levels of phosphorylated IκBα. These results are consistent with previous work that suggest HIV-1 Vpu inhibits the canonical NF-κB pathway by stabilization of IκBα. However, the authors also demonstrate that only primary Vpu stabilizes phosphorylated p105 and phosphorylated p100, thereby preventing further processing to p50 and p52, respectively, leading to the dysregulation of both canonical and non-canonical pathways. These findings further highlight that Vpu can dysregulate the NF-ĸB pathway at various points.

To identify the viral determinant in primary Vpu required for the inhibition of both NF-κB pathways, Pickering et al. mutated individual serine residues in the cytoplasmic tail and introduced naturally occurring mutations found in other primary Vpus onto the 2_87 primary Vpu background. They found that while mutations to the serine residues had the greatest effect on NF-κB inhibition, the other mutations only had a partial effect. Additionally, the authors demonstrate that only mutation of the serine at residue 57 abolished binding to β-TrCP by primary Vpu, but mutations at either serine residue 52 or 56 abolished binding by NL4.3 Vpu. They also show that this difference is not due to differing phosphorylation states between the two Vpus. These results suggest that while HIV-1 Vpu predominantly inhibits NF-κB signaling through interactions with β-TrCP, there may exist other mechanisms by which Vpu is able to suppress this pathway. In future studies, it will be of interest to determine what feature of NL4.3 Vpu accounts for the reduced potency of NF-κB inhibition.

The role of viral proteins in modulating NF-κB signaling is an area of intense research. Viruses have evolved a variety of strategies to regulate NF-κB. In addition to HIV-1, other viral families also exploit β-TrCP to inhibit NF-κB (10). By directing the ubiquitination and degradation of key regulators in the NF-κB pathway, the two paralogs of β-TrCP1 and β-TrCP2 are essential for both canonical and non-canonical NF-κB pathways. Of note, both β-TrCP paralogs were previously thought to be functionally redundant due to their lack of selectivity in the recognition of substrates. However, recent findings including those in this report indicate that the β-TrCP paralogs have selectivity for unique substrates. Further studies to understand how the two paralogs of β-TrCP differentially regulate cellular processes in the context of infection will prove highly informative for our understanding of these complex interactions.

Despite recent advances in treatments for HIV/AIDS, HIV-1 remains a global health burden. Even for patients undergoing antiretroviral therapy, HIV-1 persists in reservoirs of latently infected cells, providing a major obstacle to HIV-1 eradication. Many eradication strategies under development involve latency-reversing agents that target the activation of the non-canonical pathway. The findings by Pickering et al. indicate that this approach may prove ineffective due to the inhibitory effects of HIV-1 Vpu. Appreciation of the role of HIV-1 Vpu-meditated regulation of the NF-κB pathway will therefore inform the development of novel antivirals.
